# Evaluation of the “Foundations in Knowledge Translation” training initiative: preparing end users to practice KT

**DOI:** 10.1186/s13012-018-0755-4

**Published:** 2018-04-25

**Authors:** Jamie S. Park, Julia E. Moore, Radha Sayal, Bev J. Holmes, Gayle Scarrow, Ian D. Graham, Lianne Jeffs, Caitlyn Timmings, Shusmita Rashid, Alekhya Mascarenhas Johnson, Sharon E. Straus

**Affiliations:** 1 Li Ka Shing Knowledge Institute, St. Michael’s Hospital, Toronto, Ontario Canada; 2 Michael Smith Foundation for Health Research, Vancouver, British Columbia Canada; 3 University of Ottawa, Ottawa, Ontario Canada; 4 University of Toronto, Toronto, Ontario Canada

**Keywords:** Evaluation, Knowledge translation, Capacity building, Mixed methods, Longitudinal, Education, Implementation, Knowledge, Self-efficacy, Behavior change

## Abstract

**Background:**

Current knowledge translation (KT) training initiatives are primarily focused on preparing researchers to conduct KT research rather than on teaching KT practice to end users. Furthermore, training initiatives that focus on KT practice have not been rigorously evaluated and have focused on assessing short-term outcomes and participant satisfaction only. Thus, there is a need for longitudinal training evaluations that assess the sustainability of training outcomes and contextual factors that may influence outcomes.

**Methods:**

We evaluated the KT training initiative “Foundations in KT” using a mixed-methods longitudinal design. “Foundations in KT” provided training in KT practice and included three tailored in-person workshops, coaching, and an online platform for training materials and knowledge exchange. Two cohorts were included in the study (62 participants, including 46 “Foundations in KT” participants from 16 project teams and 16 decision-maker partners). Participants completed self-report questionnaires, focus groups, and interviews at baseline and at 6, 12, 18, and 24 months after the first workshop.

**Results:**

Participant-level outcomes include survey results which indicated that participants’ self-efficacy in evidence-based practice (*F*(1,8.9) = 23.7, *p* = 0.001, *n* = 45), KT activities (*F*(1,23.9) = 43.2, *p* < 0.001, *n* = 45), and using evidence to inform practice increased over time (*F*(1,11.0) = 6.0, *p* = 0.03, *n* = 45). Interviews and focus groups illustrated that participants’ understanding of and confidence in using KT increased from baseline to 24 months after the workshop. Interviews and focus groups suggested that the training initiative helped participants achieve their KT project objectives, plan their projects, and solve problems over time. Contextual factors include teams with high self-reported organizational capacity and commitment to implement at the start of their project had buy-in from upper management that resulted in secured funding and resources for their project. Training initiative outcomes include participants who applied the KT knowledge and skills they learned to other projects by sharing their knowledge informally with coworkers. Sustained spread of KT practice was observed with five teams at 24 months.

**Conclusions:**

We completed a longitudinal evaluation of a KT training initiative. Positive participant outcomes were sustained until 24 months after the initial workshop. Given the emphasis on implementing evidence and the need to train implementers, these findings are promising for future KT training.

**Electronic supplementary material:**

The online version of this article (10.1186/s13012-018-0755-4) contains supplementary material, which is available to authorized users.

## Background

Capacity building in the science (i.e., discovery) and practice (i.e., application) of knowledge translation (KT) is a critical element for optimizing system change. Several KT training initiatives focus on preparing researchers to conduct KT science [1–3] or secure KT grant funding [4, 5]. Alongside this training in KT science and grant writing, there is a need to build capacity among those responsible for KT practice so that clinical interventions are optimally implemented to improve patient outcomes [6]. Given that KT practice requires the involvement of various knowledge users, including patients, caregivers, clinicians, managers, and policy makers, training efforts should target all of these knowledge users and be delivered in a team environment to facilitate potential collaboration [7].

Although KT training initiatives have expanded in recent years, there are few studies evaluating their impact and fewer that evaluate their impact on individuals and organizations [8]. Evaluation of KT training activities should move beyond individual outcomes (e.g., participant satisfaction) to assess knowledge use, sustainability of training for participants, KT outcomes, and the contextual factors (e.g., an individual’s role and the organizational environment) that affect these outcomes. Application of the knowledge and skills learned in KT training initiatives (i.e., using research evidence in practice) can take time given the timelines for completing KT projects and their complexity. Thus, it is critical to assess the impact of training over time [9].

We developed “Foundations in KT” using an integrated KT approach [10] and with a partnership of knowledge users from the Michael Smith Foundation for Health Research (MSFHR; the provincial research funding agency of British Columbia [BC]), the Vancouver Coastal Health Research Institute (VCHRI), and KT scientists. The initiative was designed to provide intensive training in KT practice and was developed in response to knowledge users’ needs. We identified two levels of knowledge users: the participants in the “Foundations in KT” training initiative and their decision-maker partners (DMPs) at the micro level and both MSFHR and VCHRI at the macro level. The aim of this paper is to describe the evaluation of our KT training initiative at the micro level, specifically on participant outcomes (e.g., knowledge, self-efficacy, KT practice, and research utilization) over 2 years. We also evaluated the process for delivering the training initiative and the contextual factors that may have contributed to participants’ KT practice success. Results of the evaluation have been used for our ongoing KT training initiatives [11].

## Methods

### Study design and participants

**Fig. 1:**
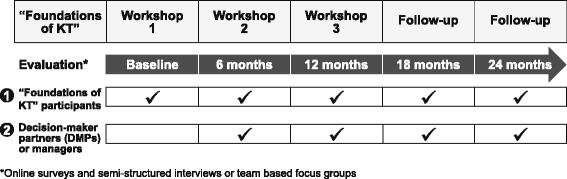
“Foundations in KT” used a mixed-methods longitudinal study design. Interviews, focus groups, and surveys were administered to “Foundations in KT” participants and decision-maker partners at baseline (only “Foundations in KT” participants) and 6, 12, 18, and 24 months after the first workshop

We used a mixed-methods (surveys, focus groups, and interviews), longitudinal study design to conduct an outcome and process evaluation of the intervention. Two groups of participants were recruited for the evaluation: (1) “Foundations in KT” participants and (2) their decision-maker partners or managers. Data were collected from two cohorts (2013–2015 and 2014–2016) of the “Foundations in KT” training initiative. Cohort 1 began the training initiative in March 2013 and cohort 2 enrolled in April 2014. Two small cohorts were used because there was substantial interest in this training and we wanted to ensure that participants received sufficient support from course facilitators for their KT projects. “Foundations in KT” participants completed self-reported questionnaires and team-based focus groups and DMPs completed self-reported questionnaires and interviews at baseline (only “Foundations in KT” participants), 6, 12, 18, and 24 months. Figure 1 presents the study design. The study was jointly coordinated by the KT Program at St. Michael’s Hospital (SMH) and MSFHR.

### Recruitment

The “Foundations in KT” training initiative was advertised in two rounds (2013 and 2014) through MSFHR and VCHRI. Recruitment emails were sent to Vancouver General Hospital, University of British Columbia (UBC) Hospital, George Frederick Strong Rehabilitation Hospital, Richmond Hospital, Lions Gate Hospital, Community Health Services for the Sunshine Coast and Bella Coola, Providence Health Centre - St. Paul’s Hospital, Rural Health Research Centre, UBC, Simon Fraser University, University of Victoria, and BC Institute of Technology. Eligible participants included clinicians, researchers, health care managers, and policy makers. If a DMP from the participant’s organization was not taking part in the training initiative, one was asked to provide a letter of support for the implementation project. Eligible DMPs were managers, policy analysts, and policy makers whose support was required to facilitate project completion. Interested participants were invited to apply in teams of two to four people and submit their names, the names of their DMPs, a description (one paragraph) of the identified health care/clinical challenge their project addressed with an explanation of its relevance, and a description (one to two paragraphs) of the strength of evidence they were interested in implementing to address this challenge. Two KT researchers and representatives from MSFHR and VCHRI reviewed the applications to assess their local relevance, strength of evidence for the clinical/health care intervention, and alignment with health care system priorities to improve care. Participants were excluded if there was not enough evidence to warrant completing a KT project or if the project did not focus on applying research evidence.

### “Foundations in KT” training initiative

The “Foundations in KT” initiative was designed to enhance participants’ knowledge and self-efficacy in KT practice and to help them develop and implement a KT project. The training initiative was designed, planned, and implemented by researchers, educators, and knowledge users from MSFHR, VCHRI, and SMH. Training initiative components included an initial in-person workshop, two in-person booster workshops provided 6 months apart, an online learning platform (Canvas), 2 years of coaching, and printed and online education materials. The training initiative design was informed by evidence from KT science and adult education. Table 1 provides an overview of the “Foundations in KT” educational principles. Educational content was aligned with these educational principles, best evidence on effective adult education, and stages of the knowledge-to-action (KTA) process model [12]. Focus groups were conducted with participants before each workshop to tailor the workshop content to their needs; session content was adapted based on learning needs to make the training initiative more relevant to participants (e.g., additional content on dissemination, KT funding, evaluation, and sustainability were included in course content (see Additional file 1)). The training initiative was facilitated by three KT researchers (SES, LJ, and IDG) with expertise in KT practice.

**Table 1:** “Foundations in KT” educational principles

“Foundations in KT” Educational Principles	Example
Interprofessional Collaboration [48]	• Course applicants were required to apply in teams of two to four that included both knowledge users and researchers • The integration of both stakeholder groups in the teaching and practice of KT is important so knowledge is relevant to their needs • Teams were required to have institutional support and were, therefore, asked to recruit their manager or a decision-maker partner (DMP) • Managers’ or DMP involvement was sought to help capture their perspectives of the implementation project and organizational factors that may influence sustainability, including scaling up of the project and further enhancing capacity in KT at their organization
Learning through practical application [49]	• Teams needed to be working on a project addressing a local knowledge-to-action gap as learning is enhanced when participants are given opportunities to apply knowledge in real-world settings
Range of teaching techniques [50–53]	• The course was based on active learning through small group work, interactive discussions (seminars and asynchronous discussions), and brief didactic sessions
Facilitation of social interaction [49, 54, 55]	• The course included an online platform to stimulate discussion of participants’ projects and to promote social connectivity; participants were asked to share their learning materials with others through the online platform.
Formal feedback and coaching	• Participants were assigned a coach they could reach out to for project-related questions; formal feedback on project plans was delivered during in-person sessions
Adult learning theory and assessment of learning needs	• Real-time assessments of learning needs was performed via interviews and focus groups before each workshop • Session content was adapted based on learning needs to make the training initiative more relevant to participants (e.g., additional content on dissemination, KT funding, evaluation, and sustainability were included in course content; see the Additional file 1 for workshop agendas).

### Outcomes

CHIR defines KT “as a dynamic and iterative process that includes synthesis, dissemination, exchange, and ethically sound application of knowledge (i.e., using research evidence) to improve the health services and products and strengthen the health care system” [13]. This paper outlines the evaluation of our KT training initiative by looking at participant-level outcomes, contextual factors, and training initiative outcomes.

*Participant-level outcomes* included participants’ self-efficacy in practicing KT activities (primary outcome), self-efficacy in evidence-based practice, KT knowledge, evidence use, comfort with evidence, and intention to use evidence. DMP outcomes included self-reported research evidence utilization, perceived importance of evidence use, and intention to use evidence.

*Contextual factors* included self-reported organizational factors to implement evidence-based practices and perceptions of the training completed by participants and DMPs.

*Training initiative outcomes* included participants’ self-reported progress of their KT project and of other KT activities beyond the intervention. Table 2 describes the outcomes and data collection methods.

**Table 2:** Research outcomes and data collection methods

Outcome	Respondent	Method	Time point
Self-efficacy in performing evidence-based management activities	“Foundations in KT” participants	EBM scale (4 items)	Baseline, 6 months, 12 months, 18 months, 24 months
Self-efficacy in practicing KT activities	“Foundations in KT” participants	Self-report survey (7 items) and semi-structured interviews and focus groups	Baseline, 6 months, 12 months, 18 months, 24 months
Knowledge in KT	“Foundations in KT” participants	Semi-structured interviews and focus groups	Baseline, 6 months, 12 months, 18 months, 24 months
Utilization of evidence	“Foundations in KT” participants and their DMPs	Self-report survey (1 item) and semi-structured interviews and focus groups	Baseline, 6 months, 12 months, 18 months, 24 months
Comfort with evidence	“Foundations in KT” participants and their DMPs	Self-report survey (1 item)	Baseline, 6 months, 12 months, 18 months, 24 months
Intention to use evidence	“Foundations in KT” participants and their DMPs	Self-report survey (3 items)	Baseline, 6 months, 12 months, 18 months, 24 months
Progress of KT project	“Foundations in KT” participants	Semi-structured interviews and focus groups	6 months, 12 months, 18 months, 24 months
Perceived readiness of organizational context for change	“Foundations in KT” participants and their DMPs	ORCA survey (77 items) and semi-structured interviews and focus groups [20]	Baseline, 6 months, 12 months, 18 months, 24 months
Perceived importance of evidence use	DMPs	Self-report survey (2 items)	Baseline, 6 months, 12 months, 18 months, 24 months
Perceptions of the training initiative	“Foundations in KT” participants	Semi-structured interviews and focus groups	6 months, 12 months, 18 months, 24 months

### Data collection

Quantitative data were collected using online surveys through FluidSurveys [14]. “Foundations in KT” participant surveys (Additional file 2) collected demographic information and included questions on self-efficacy in KT, self-efficacy in evidence-based practice, research utilization, comfort with evidence, and intention to use evidence. Self-efficacy in practicing KT activities was measured using a seven-item tool to reflect confidence in the ability to perform each step of the KTA process model. It was developed by the training facilitators and validated for face validity by 10 KT experts. The feedback obtained through the face validity assessment was focused on language; feedback was incorporated to explicitly relate each item to a step in the KTA process model. Self-efficacy in evidence-based practice was measured using the validated evidence-based medicine (EBM) scale [15]. The evidence-based medicine scale was chosen because it was the only validated tool on self-efficacy in evidence-based practice and the content is not specific to medical staff. Intention to use evidence was measured using a 3-item instrument developed for policy makers [16]. Questions for the self-efficacy and intention to use measures were reviewed to make sure the wording was relevant to the target audience and adjusted accordingly when needed. Self-reported research utilization and comfort with evidence were each measured with a single validated item that asked participants whether they have applied research in their practice and if they were comfortable with using evidence, respectively [17]. DMP-level surveys collected demographic information and included questions on research utilization, comfort with evidence, perceived importance of evidence use, and intention to use evidence; the surveys included the same questions as the participant surveys (Additional file 3). Perceived importance of evidence use was measured using two items that assessed whether DMPs believed that using evidence was important in practice and decision making [18, 19].

For the contextual surveys (Additional file 4), the organizational readiness to change (ORCA) measure was used. ORCA is a 77-item (5-point Likert scale) validated tool that measures organizational readiness to implement evidence-based practices in clinical settings. It operationalizes the constructs in the promoting action on research implementation in health services (PARIHS) framework [20].

Focus groups were conducted with each team that participated in the training initiative and semi-structured interviews with the relevant DMPs. Team-based focus group were used for the technique’s advantage for understanding team dynamics, eliciting more honest responses (team members can build off each other’s responses) and for optimizing use of research resources [21]. Telephone interviews/focus groups lasted 45 to 60 min. Team-based focus groups with participants were conducted at all five time points, and DMP interviews were collected at all time points except baseline. The semi-structured guide (Additional file 5) used during baseline focus groups with the teams addressed three main topics: KT knowledge, KT learning goals, and the objectives of participants’ KT project. The semi-structured guide (Additional file 6) used during team-based focus groups at 6, 12, 18, and 24 months addressed training initiative satisfaction and feedback, training initiative impact on KT learning goals, training initiative impact on KT project objectives, confidence in practicing KT, and suggestions for content to be included in the subsequent workshop. The semi-structured guide (Additional file 7) used during DMP interviews at 6, 12, 18, and 24 months addressed the DMP’s role in the KT project and organizational contextual factors. All interviews and focus groups were audiorecorded and transcribed; transcripts comprised the primary source of data. Quantitative and qualitative data were collected concurrently to converge findings.

The process evaluation for the intervention included collecting data from participant application forms, facilitator notes from in-person discourse during workshops, email correspondence, and Canvas discussion boards over the study period to conduct a document review.

### Analysis

Quantitative and qualitative data from the cohorts were aggregated. Quantitative analyses were conducted using SPSS v20 [22]; sum scores were calculated for outcomes that contained more than two items and for ORCA subscales. To determine if participant outcomes differed over time, multilevel modeling (MLM) was used with an unstructured covariance matrix. MLM was selected because sample sizes for each time point varied. We used a maximum likelihood estimation model, which involved estimating multiple models to determine the correct error structure using a two-level model with “time” at level 1 and “between-person variance” at level 2; models were chosen based on the Schwarz’s Bayesian Criterion. Qualitative analyses were conducted with QSR NVivo v10 [23] using a double-coded thematic analysis approach [24]. Two independent research coordinators with qualitative expertise read a portion of the interview and focus group transcripts and developed an initial coding tree. The codebook was systematically applied to the remainder of the transcripts and if any emergent themes appeared in the data, the coding tree was expanded. Inter-rater reliability between the research coordinators was calculated using Cohen’s kappa [25]. Any coding discrepancies between − 1 and 0.6 were discussed and resolved. Inter-rater reliability and coding discrepancies were used as a tool to facilitate the iterative nature of qualitative analysis and improve the fit and application of the coding tree to the data. Once data were coded, charting and visualization tools in NVivo were used to further explore the data and perform a network analysis to arrange nodes and sub-nodes into basic themes, organizing themes, and global themes [26]. A multi-source triangulation approach was used to compare quantitative and qualitative data through a meta-matrix to see which findings converged or diverged. Contextual secondary data was then examined through a document review to better understand potential reasons for why findings converged or diverged [27]. For the document review, all documents were independently coded by one coder. Following familiarization with the data, the analyst generated a list of initial codes, identified themes among the list of codes, and developed a thematic framework of analysis [28]. The analyst then reviewed the thematic framework with the project team and reviewed and synthesized abstracted data according to the major themes. Gender differences were identified as potential subgroups for analyses. As the document review was used to gather contextual data rather than primary outcome data, the documents were independently coded by one coder; however, the thematic framework was validated through team discussions before application to data to reduce bias.

### Ethics approval

Ethics approval was obtained from SMH (#12-313), UBC (#H12-02451), and VCHRI (#V12-02451) research ethics boards. Written consent to participate in the training initiative evaluation was obtained from participants and DMPs before the initiative began. Participation in the research evaluation was voluntary and no monetary compensation was awarded.

## Results

### Participants

A total of 46 participants (16 teams ranging in size from 2 to 4 people) enrolled in the “Foundations in KT” training initiative, and 16 DMPs consented to participate in the evaluation; 43 participants and 8 DMPs completed baseline surveys. All participants consented to participate in the evaluation. There were no statistically significant differences in participant demographics between cohorts 1 and 2 (Table 3) identified from the baseline survey. The majority of training initiative participants (70%, *n =* 30) and their DMPs (88%, *n =* 7) were female. Participants were evenly distributed in early-, mid-, and senior-level roles and were from a variety of research and clinical settings. Attrition increased over time; by month 24, 18 participants (39%) and 4 (25%) DMPs had withdrawn from the study due to changes in their organizational role or employment status. There was no significant difference in attrition in the subgroup analyses between male vs. female participants and DMPs. Document review and interview/focus group data illustrated that teams that had lower organizational support (e.g., lack of resources or time during work hours) appeared to drop out more frequently. In addition, some of the attrition could be attributed to missing data (e.g., the participant did not take part in 6-month data collection, but participated in 18 months or took part in the survey, but not the interview).

**Table 3:** Baseline survey data of participant and DMP demographics

		Baseline survey	
Demographic criteria		Participant (*n*)	DMP (*n*)
Total		43	8
Gender	Female	33	7
	Male	8	1
	Prefer not to disclose	2	0
Years in role	Less than 1 year	10	2
	1–2 years	9	1
	3–5 years	5	3
	6–10 years	11	1
	More than 10 years	8	1
Work setting^a^	Hospital acute	16	2
	Hospital LTC	5	0
	Hospital rehab	13	2
	Community acute	4	2
	Community LTC	5	5
	Community rehab	6	6
	Private practice acute	2	0
	Private practice LTC	1	0
	Private practice rehab	4	2
	Research acute	15	2
	Research LTC	10	1
	Research rehab	16	2
Position^a^	Clinician	12	1
	Manager	7	2
	Educator	10	1
	Researcher	18	2
	Other	16	4
Team size	Teams of two people	7	N/A
	Teams of three people	4	N/A
	Teams of four people	5	N/A

^a^Demographic grouping was not mutually exclusive

### Response rates

Survey response rates among participants were 93% (*n* = 43) at baseline, 52% (*n* = 24) at 6 months, 39% (*n* = 18) at 12 months, 41% (*n* = 19) at 18 months, and 30% (*n* = 14) at 24 months. Response rates among DMPs were 50% (*n* = 8) at baseline, 44% (*n* = 7) at 6 months, 25% (*n* = 4) at 12 months, 13% (*n* = 2) at 18 months, and 19% (*n* = 3) at 24 months. A total of 85 team focus groups and DMP interviews, which included two to three participants each, were conducted: 20 at baseline, 22 at 6 months, 16 at 12 months, 17 at 18 months, and 10 at 24 months.

### Participant-level outcomes: effect on participants’ self-efficacy and knowledge

Overall, participants’ self-efficacy in evidence-based practice (*F*(1,8.9) = 23.7, *p* = 0.001, *n* = 45) and in KT activities (*F*(1,23.9) = 43.2, *p* < 0.001, *n =* 45) increased over time (Table 4). No differences were identified by gender. In addition, participants reported an increase in comfort in using evidence to inform their practice over time (*F*(1,11.0) = 6.0, *p* = 0.03, *n =* 45). These results were also identified in the qualitative analysis; the interview data revealed that the training initiative helped participants enhance their understanding of KT and build confidence in KT practice.

**Table 4:** Participant-level outcomes and contextual factors of “Foundations in KT” participants and DMPs

Respondent	Survey measure	Test of fixed effects from baseline to 24 months	Intercept	Estimate	Standard error	*p* value
Participant-level outcomes						
Participant (*n =* 45)	Self-efficacy in the practice of evidence-based management activities (Likert 1–7)	*F*(1,8.9) = 23.7	5.1	0.02	0.004	0.001*
	Self-efficacy in the practice of KT activities (Likert 1–7)	*F*(1,23.9) = 43.2	4.4	0.04	0.006	< 0.001*
	Intent to use evidence (Likert 1–7)	*F*(1,22.1) = 0.22	6.3	−0.002	0.005	0.64
	Research utilization (Likert 1–7)	*F*(1,17.9) = 0.13	6.3	− 0.002	0.005	0.73
	Comfort with evidence (Likert 1–7)	*F*(1,11.0) = 6.0	6.0	0.01	0.004	0.03*
DMP (*n =* 12)	Intent to use evidence (Likert 1–7)	*F*(1,27.9) = 2.9	6.7	−0.12	0.07	0.10
	Research utilization (Likert 1–7)	*F*(1,20.0) = 0.19	6.6	−0.05	0.11	0.67
	Comfort with evidence (Likert 1–7)	*F*(1,27.6) = 3.5	7.0	−0.23	0.12	0.07
	Importance of evidence in practice (Likert 1–7)	*F*(1,25.7) = 1.7	6.9	−0.09	0.07	0.20
	Importance of evidence in decision making (Likert 1–7)	*F*(1,19.1) = 0.78	7.0	−0.06	0.07	0.39
Contextual factors						
Participant (*n =* 49)	ORCA- evidence subscale (Likert 1–5)	*F*(1,108.1) = 0.46	4.1	−0.02	0.03	0.50
	ORCA- context subscale (Likert 1–5)	*F*(1,114.0) = 0.05	3.7	−0.01	0.04	0.82
	ORCA- facilitation subscale (Likert 1–5)	*F*(1,110.2) = 0.42	3.7	0.02	0.04	0.52
DMP (*n =* 8)	ORCA- evidence subscale (Likert 1–5)	*F*(1,22.0) = 3.6	4.4	−0.13	0.07	0.07
	ORCA- context subscale (Likert 1–5)	*F*(1,18.2) = 0.12	3.8	0.02	0.06	0.73
	ORCA- facilitation subscale (Likert 1–5)	*F*(1,25.6) = 0.84	4.2	−0.07	0.07	0.37

Except for two teams who reported that they actively used KT in their jobs, participants joined the training initiative with no or very little knowledge about KT. At baseline, most participants understood KT as an activity that helps to translate research findings into practice and thought of it as “dissemination” or “end of grant activity”:

*I know very little about KT, and I have read the Canadian Institutes of Health Research (CIHR) definition of it a few times, and that has been somewhat illuminating and somewhat mystifying…*—Cohort 1 Team 1, Baseline

As the training initiative progressed (i.e., at 6 and 12 months), participants reported that KT was no longer an abstract concept. They described having a deeper understanding of what KT meant and knowing how to build and implement a KT plan:

*Just participating in the workshop has improved my knowledge, in general, around KT and what it means and what’s involved in it and giving me some great tools to be able to facilitate that.*—Cohort 1 Team 9, 6 months

At 18 and 24 months, participants shifted from speaking about gains in knowledge to also speaking about an increase in confidence in practicing and using KT in their current KT projects, especially because they had access to KT resources from the training initiative:

*It’s kind of made me a KT ambassador in our own unit where I feel that we should be using it to do everything, but I feel like I got a better understanding, I feel like I know the terms a little bit better, I feel that I can participate in conversations regarding policies and procedures at a better depth now than I could prior, and that’s huge, just to feel like I got the language*—Cohort 1 Team 6, 18 months

*I feel a lot more confident when I’m talking about knowledge translation of actually understanding what the various steps and differences between it all is, rather than just using an umbrella term.*—Cohort 2 Team 3, 18 months

### Participant-level outcomes: intentions to use KT and effect on participants’ KT projects

Participants’ intention to use evidence in their work (*F*(1,22.1) = 0.22, *p* = 0.64, *n* = 45) and their current use of research (*F*(1,17.9) = 0.13, *p* = 0.73, *n =* 4) was high at baseline and did not statistically change over time; there were no significant differences by gender. Focus groups suggested that the training initiative helped participants move their KT project forward and facilitated project planning and troubleshooting. Over the duration of the training initiative, participants applied the knowledge and skills they obtained and made changes in how they operationalized their project goal, including making changes to their KT strategies. Participants indicated that the goal setting activity and instructor feedback helped them re-evaluate the feasibility of their original project plans and redefine their project scope as needed. A common challenge experienced at the training initiative onset was that teams had made their project scope too large given the time and resources they had available:

*… before coming to the workshop we kind of had an idea of what our project was going to be about, and then [the workshop] gave us an opportunity to refine what our topic was and how we were going to look at making it happen.*—Cohort 1 Team 9, 6 months

As participants worked through the training initiative, they shared project updates, successes, and challenges every 6 months over an 18-month period. Participants stated that the workshops motivated them and helped them maintain project momentum because they were given adequate time to work through the KTA cycle for their project. For example, in cohort 1, Team 3 indicated that they had performed a needs assessment at 6 months, developed an intervention at 12 months, evaluated the intervention at 18 months, and were planning for dissemination and applying for additional resources to sustain the project by 24 months. Similarly, Team 6 expressed that they had completed a needs assessment at 6 months; adapted the knowledge, assessed barriers and facilitators, and implemented interventions at 12 months; monitored knowledge use at 18 months; and were in the evaluation and sustainability stages at 24 months. Having to provide project updates during each in-person workshop helped some teams adhere to their project deadlines.

Common factors that influenced project progress included project scope and complexity (i.e., number of project components and ease of subject matter comprehension).

### Other outcomes: applying KT beyond the training initiative and KT project

Qualitative findings revealed that some participants applied KT knowledge and skills obtained from the training initiative to other projects in their organization. As teams progressed from baseline to 24 months after the workshop, participants described how their new understanding of KT enabled them to identify opportunities to integrate KT in their organization, moving beyond their initial learning goals. For example, teams described spreading their knowledge by teaching at workshops or during webinars, providing informal consultations to coworkers and other stakeholders, and creating a KT product or tool that was widely disseminated. Five teams participated in majority of data collection efforts (i.e., minimal missing data); for these teams, a sustained spread of KT practice was seen at 24 months.

I didn’t realize that I could participate until I was sitting at a table for a project and I realized that KT would probably fit in perfectly with the project that they were trying to develop, because I was the only one that kind of brought it forward, I kind of took the lead on that and brought the KT framework in and that was when I really realized that was all because of me, and it was because of that workshop.—Cohort 1 Team 6, 24 months.

Participants described how the training initiative stimulated them to think more critically about the way research was conducted at their organization. Reflections at 18 and 24 months indicated that the knowledge they gained from the training initiative had better enabled them to more effectively integrate KT into grant applications. As a result, three teams from cohort 1 were successful in receiving external grant funding, including one that received a peer-reviewed, national grant. In addition, two teams received funding from their organizations and attributed much of their success in doing so to the training initiative while another team engaged training facilitators for a new project to implement a clinical toolkit in hospitals. Of the seven teams that responded at 24 months, all reported that they had achieved project objectives, worked through the KTA cycle, and disseminated the results of their KT projects at conferences, in academic journals, in newsletters, or through internal presentations:

*It’s allowed me to be a lot more critical when I see other dissemination plans, and implementation… you know, like, in implementation reviews, and stuff like that, I can be a lot more critical of methodology, and what they’ve done*—Cohort 2 Team 2, 18 months

*Participating in the project allowed us to connect within our work environment, make new connections, generate ideas, and support one another as we moved on to new projects and roles. The principles of KT are widely applicable across setting and roles and we continue to apply them in our daily work.*—Cohort 1 Team 6, 24 months

Participants expressed interest in future learning and training opportunities in KT, specifically in learning more about implementing, evaluating, and sustaining KT projects. Three of seven teams participated in additional KT training in the form of courses, workshops, and graduate studies.

### Contextual factors: effect of organizational factors on participant outcomes and KT projects

Survey data indicated that DMPs endorsed using evidence in practice and decision making and that this perception was sustained over time (*F*(1,25.7) = 1.73, *p =* 0.20, *n* = 12*, F*(1,19.1) = 0.78, *p =* 0.39, *n* = 12; Table 3). Data from the ORCA (Table 3) indicated that scores remained high in the evidence, context, and facilitation areas over time. During focus groups, training participants were asked about their capacity to implement their KT project in their organization. Teams who reported having high organizational capacity described having funding for the project, paid time to work on the project, and commitment from management to implement. Management supported these projects by providing funding and human resources because there was alignment between project objectives and the organization’s strategic direction. Most teams reported that their organization had a high level of commitment to KT as it was incorporated into their organization’s research mandate and there was support for employees to gain capacity in KT through training opportunities, such as the “Foundations in KT” initiative. In these organizations, attitudes of frontline clinical staff toward the KT projects were perceived to be positive, with staff showing an interest in project progress and a willingness to participate in project activities or dedicate time to help the project progress:

*I’d also say that another facilitator for us is a really supportive leadership at our centre around KT for not only expertise, but also just support, and understanding how important it is.*—Cohort 2 Team 3, 6 months

*I think I was actually expecting a lot more push back or more barriers from staff and I was surprise to get a lot of feedback or to get people to email us questions back about our updates. Like people actually truly seemed to be engaged in it.*—Cohort 1 Team 1, 12 months

Teams who reported having a moderate level of organizational capacity (e.g., receiving managerial support but limited funding and human resources) to implement their KT project described having buy-in from their supervisors. However, these supervisors did not have the authority to redirect resources for project use. A few teams described how over time their organization embraced KT and increased their focus on it:

*Having the support of our immediate manager has been great; however our manager unfortunately doesn’t hold budgets which is why we’re not having so much success getting people released to participate in things.*—Cohort 1 Team 8, 18 months

*I think as a whole, the institution is more focused on the nuts and bolts research. It’s only been recently that knowledge translation has gotten more of a focus. I would say it’s not the primary priority but it is around.*—Cohort 1 Team 4, 12 months

Teams who reported having low organizational capacity (e.g., little to no paid time to work on the project or available resources) described that it was difficult to achieve project progress without a supportive organizational structure that provided adequate time to work on the project and accountability to achieve milestones and deadlines. A few teams detected positive attitudes toward the project among point-of-care staff, although this enthusiasm did not necessarily translate into engagement in project activities or dedicated time to help with the project:

*I really felt the lack of institutional backing in our case… you realize that you need someone at a higher level to give you creditability for people to want to do things. We didn’t have a manager for most of it, our decision-maker changed three times, which is very difficult because you don’t who to go to for that support.*—Cohort 1 Team 6, 18 months

*… my understanding is that my coworkers think it’s a very good idea for a project…. However, I think that there are challenges that come up in coworkers when there’s not the best understanding of what it would actually take to do the project properly…*—Cohort 1 Team 2, 12 months

Participants identified several organizational factors that influenced project progress, including buy-in from external or internal stakeholders, available financial and human resources, competing priorities at the institutional level and individual level, degree of initial planning, degree to which the project topic area fit with the strategic direction of the institution and the larger research climate, and accountability structure within teams for project deliverables and milestones.

### Process evaluation of the “Foundations in KT” training initiative

The tailoring of the workshops to participants’ needs was described by participants as having a positive impact on their engagement and knowledge. In addition, having the opportunity to contribute to and collaborate on the workshop agendas allowed participants to benefit from content that was directly applicable to their project stage and learning needs. Participants perceived that success in completing their KT projects was facilitated by access to facilitators who provided ongoing technical assistance in the form of coaching and setting learning goals. Having access to course facilitators over the two-year period helped motivate and energize participants to complete project milestones. Participants mentioned that it was encouraging to meet and listen to other participants to hear about their projects, common challenges they faced, and their successes. Having opportunities to meet face to face during the workshops helped to establish and sustain relationships between participants. Changes in participant job role affected response rates over time.

## Discussion

Participation in the “Foundations in KT” training initiative was associated with increased self-efficacy and knowledge in KT practice, and this change was sustained over 24 months for some teams. Most participants identified that their baseline knowledge of KT consisted of “end of grant” KT (i.e., dissemination) and that by participating in the training initiative, they developed a deeper understanding of KT practice. At 6 and 12 months, some participants identified that having a greater understanding of how to plan for implementation made them reassess the feasibility of their project goals. Their increase in knowledge also changed their approach to project challenges and allowed them to overcome implementation roadblocks. By 18 and 24 months, participants described an increase in confidence in planning and executing KT activities and in re-imagining their role as a KT lead in their organization. This increase in applied knowledge may have influenced the progress of their KT project because they were able to re-examine its feasibility and scope. Participants stated that the length of the training initiative (i.e., 24 months) allowed them to work through the KTA cycle and apply it directly to their project. Having to provide project updates every 6 months at an in-person workshop enhanced their accountability. Participants also described significant sustained increases in self-efficacy over time. The workshop was very focused on linking concepts to participants’ projects and directly applying KT concepts; using these kinds of experiential learning techniques, for example, problem-based scenarios, can maximize skill transfer and therefore enhance self-efficacy [29, 30]. This finding is in line with other recent evaluation of training in implementation, where participants reported increased in perceived skills [31].

Our findings highlight the importance of conducting longitudinal evaluations of training initiatives because key participant-level outcomes, such as behavior change and applied knowledge, were not observed until 18 and 24 months [9]. In our study, this included detecting that the training initiative stimulated KT activities beyond participants’ initial KT projects, thereby enhancing organizational capacity. For example, some participants described applying KT concepts and tools from the training initiative to other projects. This spread of KT practice was based on participants championing KT activities and becoming a KT resource for their team or organization. Participants’ role in the organization (e.g., having a KT-specific role) was related to their ability to support organizational efforts to embrace KT. Our subsequent KT training activities explicitly asked participants about their role in the organization and prioritized the inclusion of professionals with a KT role. Participants also described developing skills to integrate KT into grant proposals by month 24, which may have contributed to the successful funding obtained for the five projects that we observed in this study. Participants also reported having career development opportunities in KT by participating in additional KT courses, workshops, and graduate studies. Future training initiatives should expand outcomes assessments to consider this “spillover effect.”

While there were several positive outcomes, there was a significant amount of attrition and some of the outcomes only emerged as significant at later time points (e.g., behavior change and applying knowledge at 18 and 24 months). Although we do not have data to document participants’ level of engagement, we hypothesize that participants whose role was related to KT and who were engaged in the workshop and subsequent activities experienced greater gains in knowledge and self-efficacy than those who were less engaged and that these participants were then more likely to continue to complete surveys over time. Unfortunately, there were no significant changes on any of the DMP or organizational readiness outcomes. It is not surprising that there were no significant changes on DMP outcomes, since they did not directly attend any training; however, we hoped by using a team-based learning approach and engaging the DMPs, there would be an impact on organizational readiness. More intensive organization level interventions may be necessary to enhance readiness [32].

Currently, there is a lack of evidence supporting the effectiveness of KT training initiatives, and most of the literature has focused on evaluating training in KT science [2, 5, 33, 34]. To our knowledge, our study is the first longitudinal evaluation of a training initiative focused on KT practice; as such, it is unique in providing evidence of an increase in knowledge and self-efficacy related to KT practice beyond the initial workshop and of the spread of KT knowledge and skills to other activities conducted by participants. Extending skills and training to other KT projects has the potential to build organizational capacity. We have directly used the findings from this evaluation for our ongoing KT trainings, including KT Basics, a 2 day workshop on the basics of applying KT and Practicing KT, a longitudinal, comprehensive training supporting health care professionals to apply KT to their own work [11]. We have delivered KT Basics to three cohorts from 2013 to 2017 and practicing KT seven times in five countries (Canada, Australia, Uganda, Ethiopia, Tanzania).

Integration of leadership support, in the form of DMPs who were engaged as part of the training model, was a facilitator for KT project success within organizations. Previous studies have shown the importance of leadership for championing organizational learning climates and creating readiness for change [35, 36]. The integration of DMPs was used as a strategy to foster a closer link between teams and their leadership and to help resolve perceptual differences in project goals, which is linked to better performance [37, 38]. In our study, leadership support to facilitate access to resources and organizational commitment to KT was perceived to be an ideal scenario for KT practice [37–39]. High organizational support included buy-in from management and a strategic alignment between organizations’ core values and KT project goals. In comparison, low organizational support and commitment was a barrier to KT project progress, and our process evaluation identified that changes in job role and scope led to withdrawal from the training initiative. These results are consistent with literature that highlights the impact of supportive leadership and organizational culture and climate on successful implementation [40, 41]. Research has shown that leadership predicts the successful implementation of innovations in health care and beyond [42, 43]. Additionally, organizational leadership is critical for securing and designating resources and reinforcing policies for implementation, leading to better sustainability of project outcomes [44–47].

Some limitations should be noted. First, not all participants completed the surveys, focus groups, or interviews. It is possible that participants who were less engaged in the training did not participate in the assessment and, as a result, their perspectives and outcomes were not captured. Additionally, DMPs did not complete interviews at baseline and although, focus groups can be advantageous for understanding a common experience, participants may have been hesitant to express negative experiences with other team members in the group data collection setting. Second, our data were collected using self-report measures, some of which had not been assessed for validity. However, the survey results were consistent with the interviews and focus group results. Third, we used a quasi-experimental research design without the use of a control group. For this reason, causal inferences regarding predictors and outcomes cannot be made. Fourth, the study was conducted in a single health system within a province and therefore may not be generalizable to other settings; however, this is the largest and most populous province.

Strengths of this study include the use of mixed methods, inclusion of both participants and DMPs, tailoring of the training initiative to participants’ needs, and the duration of the follow-up period. Additional strengths include the use of an integrated KT approach whereby the knowledge users (researcher funders and managers in BC) were involved in the development of the course, which was designed and delivered in response to local needs.

## Conclusions

Tailored training increased participant knowledge and self-efficacy in KT practice. This change may have influenced the progress of participants’ KT projects and helped spread KT within organizations. Contextual factors that were perceived to affect outcomes included organizational factors, such as leadership and organizational commitment, and participant factors, such as job role and scope.

## Additional files

Additional file 1: Workshop agendas. (DOCX 36 kb)

Additional file 2: Foundations in KT participant-level surveys. (DOCX 101 kb)

Additional file 3: DMP-level surveys. (DOCX 22 kb)

Additional file 4: Contextual-level surveys. (DOCX 279 kb)

Additional file 5: Baseline semi-structured interview guide for Foundations in KT participants. (DOCX 18 kb)

Additional file 6: 6, 12, 18, and 24 month semi-structured interview guide for Foundations in KT participants. (DOCX 24 kb)

Additional file 7: 6, 12, 18, and 24 month semi-structured interview guide for DMPs. (DOCX 24 kb)

## Abbreviations

BC: British Columbia
CIHR: Canadian Institutes of Health Research
DMPs: Decision-maker partners
EBM: Evidence-based medicine
KT: Knowledge translation
KTA: Knowledge to action
MSFHR: Michael Smith Foundation for Health Research
ORCA: Organizational Readiness to Change Assessment
SMH: St. Michael’s Hospital
UBC: University of British Columbia
VCHRI: Vancouver Coastal Health Research Institute
