# Supplementary material for: Evaluation of the “Foundations in Knowledge Translation” training initiative: preparing end users to practice KT
Source: Implement Sci. 2018 Apr 25;13:63. doi: 10.1186/s13012-018-0755-4 (PMC5918493; doi:10.1186/s13012-018-0755-4)
Supplement: Supplementary file 1 — Workshop agendas. (DOCX 36 kb) [file 13012_2018_755_MOESM1_ESM.docx]

“Foundations of Knowledge Translation” Course

Park Inn and Suites (Oak Room), 898 West Broadway, Vancouver, BC, V5Z 1J8

**Foundations Workshop #1 DAY ONE Wednesday, March 20, 2013**

| **Session Time** | **Session Topic** |
| --- | --- |
| 8:00 AM – 9:00AM | What is KT?   - The Knowledge to Action Loop - Overview of effective strategies in KT   What is the ‘K’ in KT?   - Knowledge synthesis - Knowledge tools/products |
| 9:00 AM – 10:15 AM | Small Group Session   - Discuss evidence and rationale for your KT project |
| 10:15 AM – 10:30AM | BREAK |
| 10:30 AM – 11:15 AM | How do we identify and engage relevant stakeholders?  How do we measure the knowledge gap?   - Chart audits/administrative databases/clinical databases - Needs assessments |
| 11:15 AM – 12:00PM | Small Group Session   - Identifying relevant stakeholders and measuring the knowledge gap in context of own projects |
| 12:00 PM – 12:45PM | - LUNCH |
| 12:45 PM – 1:15 PM | Adapting knowledge to local context  Assessing barriers and facilitators to knowledge use |
| 1:15 PM – 2:15PM | Small Group Session   - Adapting the relevant knowledge and assessing barriers and facilitators to knowledge use in context of own projects |
| 2:15 PM – 2:45PM | BREAK |
| 2:45 PM – 4:00PM | Feedback and large group discussion/review of projects |
| 6:00 PM (optional, RVSP required) | Networking dinner at Rouge Kitchen and Wetbar, 602 W Broadway |

**Foundations Workshop #1 DAY TWO Thursday, March 21, 2013**

| **Session Time** | **Session Topic** |
| --- | --- |
| 8:00 AM – 8:45 AM | Select, tailor, implement KT interventions   - Developing/selecting the KT strategy/tool/intervention including feedback interventions, electronic interventions, patient mediated interventions, organizational interventions |
| 8:45 AM – 10:00 AM | Small Group Session   - Developing your KT intervention |
| 10:00AM – 10:15 AM | BREAK |
| 10:15AM – 11:15 AM | Monitoring and evaluation of the implementation of the KT Intervention   - Framework for evaluating complex interventions |
| 11:15AM – 12:15PM | Working Lunch:  Small Group Session   - Monitoring and evaluating the implementation of your KT intervention |
| 12:15PM – 12:45PM | Developing strategies to assess and optimize sustainability |
| 12:45PM – 1:30 PM | Small Group Session   - Developing a strategy for sustaining your KT interventions |
| 1:30PM – 2:00PM | Feedback and setting goals for next 6 months |

“Foundations of Knowledge Translation” Course

Lecture Hall, Blusson Spinal Cord Centre, 818 West 10th Avenue, Vancouver, BC

**Foundations Workshop #2 Wednesday, September 25, 2013**

| **Session Time** | **Session Topic** |
| --- | --- |
| 7:30 AM – 8:00AM | Breakfast available |
| 8:00 AM – 8:30 AM | Introductions and overview of objectives  Team Presentations (10 minutes per team) and Feedback (15 minutes per team) |
| 8:30 AM – 8:55 AM | Team 1 Presentation: Personalized Genomics in Primary Care |
| 8:55 AM – 9:20 AM | Team 2 Presentation: Rick Hansen Spinal Cord Injury Registry (RHSCIR) |
| 9:20 AM – 9:45 AM | Team 3 Presentation: BC Physiotherapists’ Awareness and Use of the Canadian Cervical Spine Rule in Clinical Practice |
| 9:45 AM – 10:15AM | BREAK |
| 10:15 AM – 10:45 AM | Team 4 Presentation: Optimal Birth BC |
| 10:40 AM – 11:05 AM | Team 6 Presentation: Delirium the Intensive Care Unit (ICU) |
| 11:05 AM – 11:30 AM | Team 8 Presentation: Respiratory secretion clearance treatments |
| 11:30 AM – 11:55 AM | Team 9 Presentation: Self-Management |
| 11:55 AM – 1:00 PM | LUNCH |
| 1:00 PM – 2:00 PM | Next steps |
| 2:00 PM – 4:00 PM (Optional) | Work period for teams (rooms available) |

“Foundations of Knowledge Translation” Course

Lecture Hall, Blusson Spinal Cord Centre, 818 West 10th Avenue, Vancouver, BC

**Foundations Workshop #3 Tuesday, April 8, 2014**

| **Session Time** | **Session Topic** |
| --- | --- |
| 7:30 AM – 8:00AM | Breakfast available |
| 8:00 AM – 8:10 AM | Welcome remarks |
| 8:10 AM – 9:40 AM | Team Project Updates (verbal) and Feedback [10 minutes per team for updates plus feedback] |
| 9:40 AM – 10:30 AM | Planning for Sustainability |
| 10:30 AM – 10:45 AM | BREAK (refreshments will be served) |
| 10:45 AM – 12:00 PM | Small Group Breakout Session – developing a strategy for sustaining your KT interventions |
| 12:00 PM – 1:00 PM | LUNCH (provided) |
| 1:00 PM – 1:45 PM | Strategies and Helpful Tips for Dissemination/Publication of Project Findings |
| 1:45 PM – 2:30 PM | Small Group Breakout Session – developing a dissemination plan |
| 2:30 PM – 2:45 PM | BREAK (refreshments will be served) |
| 2:45 PM – 3:30 PM | Large Group Discussion and Next Steps |
| 3:30 PM – 3:45 PM | Team Goals for the Next Six Months |
| 3:45PM – 4:00 PM | Wrap Up |

“Foundations of Knowledge Translation” Course

Lecture Hall, Blusson Spinal Cord Centre, 818 West 10th Avenue, Vancouver, BC

**Foundations Workshop #1 DAY ONE Wednesday, April 9, 2014**

| **Session Time** | **Session Topic** |
| --- | --- |
| 7:30 AM – 8:00AM | Breakfast available |
| 8:00AM – 9:00AM | What is KT?   - The Knowledge to Action Loop - Overview of effective strategies in KT   What is the ‘K’ in KT?   - Knowledge synthesis - Knowledge tools/products |
| 9:00 AM – 10:15 AM | Small Group Session   - Discuss evidence and rationale for your KT project |
| 10:15 AM – 10:30AM | BREAK (refreshments will be provided) |
| 10:30 AM – 11:15 AM | How do we identify and engage relevant stakeholders?  How do we measure the knowledge gap?   - Chart audits/administrative databases/clinical databases - Needs assessments |
| 11:15 AM – 12:00PM | Small Group Session   - Identifying relevant stakeholders and measuring the knowledge gap in context of own projects |
| 12:00 PM – 12:45PM | - LUNCH |
| 12:45 PM – 1:15 PM | Adapting knowledge to local context  Assessing barriers and facilitators to knowledge use |
| 1:15 PM – 2:15PM | Small Group Session   - Adapting the relevant knowledge and assessing barriers and facilitators to knowledge use in context of own projects |
| 2:15 PM – 2:45PM | BREAK (refreshments will be provided) |
| 2:45 PM – 4:00PM | Feedback and large group discussion/review of projects |
|  |  |

“Foundations of Knowledge Translation” Course

Lecture Hall, Blusson Spinal Cord Centre, 818 West 10th Avenue, Vancouver, BC

**Foundations Workshop #1 DAY TWO Thursday, April 10, 2014**

| **Session Time** | **Session Topic** |
| --- | --- |
| 7:30 AM – 8:00AM | Breakfast available |
| 8:00 AM – 8:45 AM | Select, tailor, implement KT interventions   - Developing/selecting the KT strategy/tool/intervention including feedback interventions, electronic interventions, patient mediated interventions, organizational interventions |
| 8:45 AM – 10:00 AM | Small Group Session   - Developing your KT intervention |
| 10:00AM – 10:15 AM | BREAK (refreshments will be provided) |
| 10:15AM – 11:15 AM | Monitoring and evaluation of the implementation of the KT Intervention   - Framework for evaluating complex interventions |
| 11:15AM – 12:15PM | Working Lunch: (Lunch will be provided)  Small Group Breakout Session   - Monitoring and evaluating the implementation of your KT intervention |
| 12:15PM – 12:45PM | Developing strategies to assess and optimize sustainability |
| 12:45PM – 1:30 PM | Small Group Breakout Session   - Developing a strategy for sustaining your KT interventions |
| 1:30PM – 1:45PM | Introduction to Canvas (online Community of Practice Forum) |
| 1:45PM – 2:30PM | Feedback and setting goals for next 6 months |

“Foundations of Knowledge Translation” Course

Lecture Hall, Blusson Spinal Cord Centre, 818 West 10th Avenue, Vancouver, BC

**Foundations Workshop #2 Wednesday, October 22, 2014**

| **Session Time** | **Session Topic** |
| --- | --- |
| 7:30 AM – 8:00AM | Breakfast available |
| 8:00 AM – 8:30 AM | Welcome and short refresher on the Knowledge to Action Cycle  **Team Updates on Progress (5 minutes per team) and Feedback (10 minutes per team)** |
| 8:30 AM – 8:45 AM | Team 1 Presentation: Integrating SMS use into clinical practice |
| 8:45 AM – 9:00 AM | Team 2 Presentation: Blood sampling practices in critical care: How critical are they? |
| 9:00 AM – 9:15 AM | Team 3 Presentation: Older adults’ mobility and physical activity |
| 9:15 AM – 9:30 AM | Team 4 Presentation: Promoting physical activity and exercise among people with spinal cord injury |
| 9:30 AM – 9:45 AM | Team 5 Presentation: Enhanced disability management program |
| 9:45 AM – 10:00 AM | BREAK |
| 10:00 AM – 11:00 AM | Case examples of KT in diverse settings and common challenges   - Examples of how KT is applied in different settings - Common barriers - Tips and strategies for addressing barriers and leveraging opportunities |
| 11:00 AM – 12:00 PM | Small Group Breakout Session #1   - Team work period to discuss strategies for successful implementation, barriers experienced to date, and strategies for overcoming barriers |
| 12:00 PM – 1:00 PM | Working lunch (Continue to work in small groups for session #1) |
| 1:00 PM – 2:15 PM | Strategies and Helpful Tips for End of Grant KT   - Opportunities for KT funding - Disseminating findings to diverse stakeholder groups |
| 2:15 PM – 3:15 PM | Small Group Breakout Session #2   - Practice sharing between teams about strategies for dissemination |
| 3:15 PM – 3:30 PM | Refresher on CANVAS   - Why it’s important - Analytics to date |
| 3:30 PM – 4:00 PM ( | Wrap up and setting goals for the next 6 months |

“Foundations of Knowledge Translation” Course

Jack Bell Research Centre, Room 237

2660 Oak Street, Vancouver, BC

***To gain access to the building, please call Amber Hay at 778-229-8119***

**Foundations Workshop #3 Friday, April 17, 2015**

| **Session Time** | **Session Topic** |
| --- | --- |
| 7:30 AM – 8:00AM | Breakfast available (optional) |
| 8:00 AM – 8:10 AM | Welcome |
| 8:10 AM – 8:5 0 AM | Team Project Updates (verbal) and Feedback [10 minutes per team for updates plus feedback] |
| 9:50 AM – 9:30 AM | Planning for Sustainability |
| 9:30 AM – 9:45 AM | BREAK (refreshments will be served) |
| 9:45 AM – 10:15 AM | Examples and Helpful Tips for Dissemination and Exchange Strategies/Activities |
| 10:15 AM – 10:30 AM | KT Project Implementation Case Examples from Cohort I – Lessons learned and key success factors   - BC Physiotherapists’ Awareness and Use of the Canadian Cervical Spine Rule in Clinical Practice |
| 10:30 AM – 11:30 AM | Small Group Breakout Session  • Developing a dissemination and exchange plan  • Developing a strategy for sustaining your KT interventions |
| 11:35 AM – 12:00 PM | KT Project Implementation Case Examples from Cohort I – Lessons learned and key success factors [15 minutes per team]  • Delirium the Intensive Care Unit (ICU)  • Self-management |
| 12:00 PM –12:45 PM | LUNCH (provided) |
| 12:45 PM – 1:15 PM | KT Resources and Future Directions of KT Science and Practice |
| 1:15 PM – 1:30 PM | Wrap up |
