# Supplementary material for: Evaluation of the “Foundations in Knowledge Translation” training initiative: preparing end users to practice KT
Source: Implement Sci. 2018 Apr 25;13:63. doi: 10.1186/s13012-018-0755-4 (PMC5918493; doi:10.1186/s13012-018-0755-4)
Supplement: Supplementary file 2 — Foundations in KT participant-level surveys. (DOCX 101 kb) [file 13012_2018_755_MOESM2_ESM.docx]

Participant Survey

# Please enter your participant ID as provided in the email invitation:

# Demographics

This section contains a series of questions about your demographic characteristics.

# 1. Please indicate your sex.

|  | Male |
| --- | --- |
|  | Female |

# 2. Please indicate your current work setting (please check all that apply).

|  | Acute Care | Long-Term Care | Rehabilitation |
| --- | --- | --- | --- |
| Hospital |  |  |  |
| Community |  |  |  |
| Private Practice |  |  |  |
| Research |  |  |  |

# 3. Please indicate your current position (please check all that apply).

|  | Clinician |
| --- | --- |
|  | Manager |
|  | Educator |
|  | Researcher |
|  | Other: ______________________ |

# 4. Please indicate the number of years you have been in your current role.

|  | Less than 1 year |
| --- | --- |
|  | 1- 2 years |
|  | 3- 5 years |
|  | More than 5 years |
|  | More than 10 years |

# This survey is designed to assess your current and intended use of evidence to inform your practice and decision-making. Please answer each question to the best of your ability.

# 1. Self-efficacy to perform evidence-based management (EBM) activities

Please rate your ability to perform each activity from 1 (terrible, I cannot do this at all) to 4 (not sure or I may be able to do this) to 7 (excellent, I can do this well).

|  | 1- Terrible | 2- Very Poor | 3- Poor | 4- Not sure | 5- Good | 6- Very good | 7- Excellent |
| --- | --- | --- | --- | --- | --- | --- | --- |
| a) Formulate a question to guide a literature search. |  |  |  |  |  |  |  |
| b) Effectively search the relevant literature for reliability and relevance. |  |  |  |  |  |  |  |
| c) Critically appraise the literature for reliability and relevance. |  |  |  |  |  |  |  |
| d) Critically appraise the strengths and weaknesses of different study designs. |  |  |  |  |  |  |  |

# 2. Self-efficacy in the practice of knowledge translation

Please rate your ability to perform each activity from 1 (terrible, I cannot do this at all) to 4 (not sure or I may be able to do this) to 7 (excellent, I can do this well).

|  | 1- Terrible | 2- Very Poor | 3- Poor | 4- Not sure | 5- Good | 6- Very good | 7- Excellent |
| --- | --- | --- | --- | --- | --- | --- | --- |
| a) I can conduct a needs assessment for knowledge users. |  |  |  |  |  |  |  |
| b) I can adapt research evidence to the needs of local knowledge users/stakeholders (e.g., clinicians, health care managers) |  |  |  |  |  |  |  |
| c) I can identify barriers and facilitators to knowledge uptake across different knowledge users. |  |  |  |  |  |  |  |
| d) I can develop an evidence-based knowledge translation intervention based on assessment of barriers and facilitators to knowledge uptake. |  |  |  |  |  |  |  |
| e) I can develop a strategy for monitoring knowledge use. |  |  |  |  |  |  |  |
| f) I can develop a strategy for evaluating relevant outcomes from knowledge use. |  |  |  |  |  |  |  |
| g) I can develop a strategy for sustaining knowledge use over time. |  |  |  |  |  |  |  |

# 3. Self-report of research utilization and comfort with evidence

Please rate your ability to perform each activity from 1 (Strongly disagree, I cannot do this at all) to 4 (neither agree or disagree) to 7 (Strongly agree, I can do this well).

|  | 1- Strongly disagree | 2- Disagree | 3- Somewhat disagree | 4- Neither agree nor disagree | 5- Somewhat agree | 6- Agree | 7- Strongly agree |
| --- | --- | --- | --- | --- | --- | --- | --- |
| a) I use evidence to inform decision-making in my current role |  |  |  |  |  |  |  |
| b) I am comfortable using evidence to inform my practice |  |  |  |  |  |  |  |

# 4. Intention to use evidence

Please rate your ability to perform each activity from 1 (Strongly disagree, I cannot do this at all) to 4 (neither agree or disagree) to 7 (Strongly agree, I can do this well).

|  | 1- Strongly disagree | 2- Disagree | 3- Somewhat disagree | 4- Neither agree nor disagree | 5- Somewhat agree | 6- Agree | 7- Strongly agree |
| --- | --- | --- | --- | --- | --- | --- | --- |
| a) I expect to use high-quality evidence to help work through what I will discuss with managers, patients or clinicians during encounters with them. |  |  |  |  |  |  |  |
| b) I want to use high-quality evidence to help work through what I will discuss with managers, patients or clinicians during encounters with them. |  |  |  |  |  |  |  |
| c) I intend to use high-quality evidence to help work through what I will discuss with managers, patients or clinicians during encounters with them. |  |  |  |  |  |  |  |

# End of survey.

Thank you for your participation. Once you have submitted your survey, you will be given the option to download and print your responses as a PDF or Word document (through the toolbar located on the top of your browser). We recommend you do this so you can compare your responses over time.
