# Supplementary material for: Evaluation of the “Foundations in Knowledge Translation” training initiative: preparing end users to practice KT
Source: Implement Sci. 2018 Apr 25;13:63. doi: 10.1186/s13012-018-0755-4 (PMC5918493; doi:10.1186/s13012-018-0755-4)
Supplement: Supplementary file 3 — DMP-level surveys. (DOCX 22 kb) [file 13012_2018_755_MOESM3_ESM.docx]

Decision-Maker Partner Survey

# Please enter your participant ID as provided in the email invitation:

# Demographics

This section contains a series of questions about your demographic characteristics.

# 1. Please indicate your sex.

|  | Male |
| --- | --- |
|  | Female |

# 2. Please indicate your current work setting (please check all that apply).

|  | Acute Care | Long-Term Care | Rehabilitation |
| --- | --- | --- | --- |
| Hospital |  |  |  |
| Community |  |  |  |
| Private Practice |  |  |  |
| Research |  |  |  |

# 3. Please indicate your current position (please check all that apply).

|  | Clinician |
| --- | --- |
|  | Manager |
|  | Educator |
|  | Researcher |
|  | Other: ______________________ |

# 4. Please indicate the number of years you have been in your current role.

|  | Less than 1 year |
| --- | --- |
|  | 1- 2 years |
|  | 3- 5 years |
|  | More than 5 years |
|  | More than 10 years |

# This survey is designed to assess your current and intended use of evidence to inform your practice and decision-making. Please answer each question to the best of your ability.

# 1. Self-report of research utilization and comfort with evidence

Please indicate your level of agreement with the following statements on a scale from 1 to 7, where 1 is strongly disagree and 7 is strongly agree.

# a) I use evidence to inform decision-making in my current role.

|  | 1- Strongly disagree | 2- Disagree | 3- Somewhat disagree | 4- Neither agree nor disagree | 5- Somewhat agree | 6- Agree | 7- Strongly agree |
| --- | --- | --- | --- | --- | --- | --- | --- |
|  |  |  |  |  |  |  |  |

# b) I am comfortable using evidence to inform my current practice.

|  | 1- Strongly disagree | 2- Disagree | 3- Somewhat disagree | 4- Neither agree nor disagree | 5- Somewhat agree | 6- Agree | 7- Strongly agree |
| --- | --- | --- | --- | --- | --- | --- | --- |
|  |  |  |  |  |  |  |  |

# 2. Perceived importance of evidence use

Please indicate your level of agreement with the following statements on a scale from 1 to 7, where 1 is strongly disagree and 7 is strongly agree.

# a) I think it is important to use evidence in practice.

|  | 1- Strongly disagree | 2- Disagree | 3- Somewhat disagree | 4- Neither agree nor disagree | 5- Somewhat agree | 6- Agree | 7- Strongly agree |
| --- | --- | --- | --- | --- | --- | --- | --- |
|  |  |  |  |  |  |  |  |

# b) I think it is important to use evidence in decision-making.

|  | 1- Strongly disagree | 2- Disagree | 3- Somewhat disagree | 4- Neither agree nor disagree | 5- Somewhat agree | 6- Agree | 7- Strongly agree |
| --- | --- | --- | --- | --- | --- | --- | --- |
|  |  |  |  |  |  |  |  |

# 3. Intention to use evidence

Please indicate your level of agreement with the following statements on a scale from 1 to 7, where 1 is strongly disagree and 7 is strongly agree.

# a) I expect to use high-quality evidence to help work through what I will discuss with managers, patients or clinicians during encounters with them.

|  | 1- Strongly disagree | 2- Disagree | 3- Somewhat disagree | 4- Neither agree nor disagree | 5- Somewhat agree | 6- Agree | 7- Strongly agree |
| --- | --- | --- | --- | --- | --- | --- | --- |
|  |  |  |  |  |  |  |  |

# b) I want to use high-quality evidence to help work through what I will discuss with managers, patients or clinicians during encounters with them.

|  | 1- Strongly disagree | 2- Disagree | 3- Somewhat disagree | 4- Neither agree nor disagree | 5- Somewhat agree | 6- Agree | 7- Strongly agree |
| --- | --- | --- | --- | --- | --- | --- | --- |
|  |  |  |  |  |  |  |  |

# c) I intend to use high-quality evidence to help work through what I will discuss with managers, patients or clinicians during encounters with them.

|  | 1- Strongly disagree | 2- Disagree | 3- Somewhat disagree | 4- Neither agree nor disagree | 5- Somewhat agree | 6- Agree | 7- Strongly agree |
| --- | --- | --- | --- | --- | --- | --- | --- |
|  |  |  |  |  |  |  |  |

# End of survey.

Thank you for your participation. Once you have submitted your survey, you will be given the option to download and print your responses as a PDF or Word document (through the toolbar located on the top of your browser). We recommend you do this so you can compare your responses over time.
