# Supplementary material for: Evaluation of the “Foundations in Knowledge Translation” training initiative: preparing end users to practice KT
Source: Implement Sci. 2018 Apr 25;13:63. doi: 10.1186/s13012-018-0755-4 (PMC5918493; doi:10.1186/s13012-018-0755-4)
Supplement: Supplementary file 4 — Contextual-level surveys. (DOCX 279 kb) [file 13012_2018_755_MOESM4_ESM.docx]

Organizational Readiness to Change Assessment

**Please enter your participant ID as provided in the email invitation:**

**This survey is used as a tool to understand readiness for change and to identify barriers or challenges to implementation. The questions assess: (I) level of evidence; (II) organizational context; and (III) facilitation of implementation.**

**I.​ Evidence Assessment**

Consider the following statement when answering the questions below: The activities of your project will improve outcomes for patients (or your target audience).

**1. Based on your assessment of the evidence basis for this statement, please rate the strength of the evidence in your opinion, on a scale of 1 to 5 where 1 is very weak evidence and 5 is very strong evidence:**

|  | 1- Very weak | 2- Weak | 3- Neither Weak nor Strong | 4- Strong | 5- Very Strong | Don't Know/Not Applicable |
| --- | --- | --- | --- | --- | --- | --- |
|  |  |  |  |  |  |  |

**2. Now, please rate the strength of the evidence basis for this statement based on how you think clinical experts (or managers or patients) in your institution feel about the strength of the evidence, on a 1 to 5 scale similar to the one above:**

|  | 1 - Very Weak | 2 - Weak | 3 - Neither Weak nor Strong | 4 - Strong | 5 - Very Strong | Don't Know/Not Applicable |
| --- | --- | --- | --- | --- | --- | --- |
|  |  |  |  |  |  |  |

**For each of the following statements, please rate the strength of your agreement with the statement, from 1 (strongly disagree) to 5 (strongly agree)**

**3. Research: The proposed practice changes or guideline implementation:**

|  | 1 - Strongly Disagree | 2 - Diagree | 3 - Neither Agree nor Disagree | 4 - Agree | 5 - Strongly Agree | Don't Know/Not Applicable |
| --- | --- | --- | --- | --- | --- | --- |
| a) are supported by randomized controlled trials (RCTs) or other scientific evidence from your organization |  |  |  |  |  |  |
| b) are supported by RCTs or other scientific evidence from other health care systems |  |  |  |  |  |  |
| c) should be effective, based on current scientific knowledge |  |  |  |  |  |  |

**4. Clinical/Practical Experience: The proposed practice changes or guideline implementation:**

|  | 1 - Strongly Disagree | 2 - Disagree | 3 - Neither Agree nor Disagree | 4 - Agree | 5 - Strongly Agree | Don't Know/Not Applicable |
| --- | --- | --- | --- | --- | --- | --- |
| a) are supported by clinical/practical experience with your patients |  |  |  |  |  |  |
| b) are supported by clinical/practical experience with patients in other health care systems |  |  |  |  |  |  |
| c) conform to the opinions of clinical/practice experts in this setting |  |  |  |  |  |  |

**5. Patient Preferences: The proposed practice changes or guideline implementation:**

|  | 1 - Strongly Disagree | 2 - Disagree | 3 - Neither Agree nor Disagree | 4 - Agree | 5 - Strongly Agree | Don't Know/Not Applicable |
| --- | --- | --- | --- | --- | --- | --- |
| a) have been well-accepted by patients in a pilot study |  |  |  |  |  |  |
| b) are consistent with clinical practices that have been accepted by patients |  |  |  |  |  |  |
| c) take into consideration the needs and preferences of patients |  |  |  |  |  |  |
| d) appear to have more advantages than disadvantages for patients |  |  |  |  |  |  |

**II.​ Context Assessment**

For each of the following statements, please rate the strength of your agreement with the statement, from 1 (strongly disagree) to 5 (strongly agree).

**6. Culture: Senior leadership/clinical management in your organization:**

|  | 1 - Strongly Disagree | 2 - Disagree | 3 - Neither Agree nor Disagree | 4 - Agree | 5 - Strongly Agree | Don't Know/Not Applicable |
| --- | --- | --- | --- | --- | --- | --- |
| a) reward clinical innovation and creativity to improve patient care |  |  |  |  |  |  |
| b) solicit opinions of clinical staff regarding decisions about patient care |  |  |  |  |  |  |
| c) seek ways to improve patient education and increase patient participation in treatment |  |  |  |  |  |  |

**7. Culture: Staff members in your organization:**

|  | 1 -Strongly Disagree | 2 - Disagree | 3 - Neither Agree nor Disagree | 4 - Agree | 5 - Strongly Agree | Don't Know/Not Applicable |
| --- | --- | --- | --- | --- | --- | --- |
| a) have a sense of personal responsibility for improving patient care and outcomes |  |  |  |  |  |  |
| b) cooperate to maintain and improve effectiveness of patient care |  |  |  |  |  |  |
| c) are willing to innovate and/or experiment to improve clinical procedures |  |  |  |  |  |  |
| d) are receptive to change in clinical processes |  |  |  |  |  |  |

**8. Leadership: Senior leadership/clinical management in your organization:**

|  | 1 - Strongly Disagree | 2 - Disagree | 3 - Neither Agree nor Disagree | 4 - Agree | 5 - Strongly Agree | Don't Know/Not Applicable |
| --- | --- | --- | --- | --- | --- | --- |
| a) provide effective management for continuous improvement of patient care |  |  |  |  |  |  |
| b) clearly define areas of responsibility and authority for clinical managers and staff |  |  |  |  |  |  |
| c) promote team building to solve clinical care problems |  |  |  |  |  |  |
| d) promote communication among clinical services and units |  |  |  |  |  |  |

**9. Measurement: Senior Leadership/clinical management in your organization:**

|  | 1 - Strongly Disagree | 2 - Disagree | 3 - Neither Agree nor Disagree | 4 - Agree | 5 - Strongly Agree | Don't Know/Not Applicable |
| --- | --- | --- | --- | --- | --- | --- |
| a) provide staff with information on performance measures and guidelines |  |  |  |  |  |  |
| b) establish clear goals for patient care processes and outcomes |  |  |  |  |  |  |
| c) provide staff members with feedback/data on effects of clinical decisions |  |  |  |  |  |  |
| d) hold staff members accountable for achieving results |  |  |  |  |  |  |

**10. Readiness for change: Opinion leaders in your organization:**

|  | 1 -Strongly Disagree | 2 - Disagree | 3 - Neither Agree nor Disagree | 4 - Agree | 5 - Strongly Agree | Don't Know/Not Applicable |
| --- | --- | --- | --- | --- | --- | --- |
| a) believe that the current practice patterns can be improved |  |  |  |  |  |  |
| b) encourage and support changes in practice patterns to improve patient care |  |  |  |  |  |  |
| c) are willing to try new clinical protocols |  |  |  |  |  |  |
| d) work cooperatively with senior leadership/clinical management to make appropriate changes |  |  |  |  |  |  |

**11. Resources: In general in my organization, when there is agreement that change needs to happen:**

|  | 1 - Strongly Disagree | 2 - Disagree | 3 - Neither Agree nor Disagree | 4 - Agree | 5 - Strongly Agree | Don't Know/Not Applicable |
| --- | --- | --- | --- | --- | --- | --- |
| a) we have the necessary support in terms of budget or financial resources |  |  |  |  |  |  |
| b) we have the necessary support in terms of training |  |  |  |  |  |  |
| c) we have the necessary support in terms of facilities |  |  |  |  |  |  |
| d) we have the necessary support in terms of staffing |  |  |  |  |  |  |

**III. Facilitation Assessment:**

For each of the following statements, please rate the strength of your agreement with the statement, from 1 (strongly disagree) to 5 (strongly agree):

**12. Characteristics: Senior leadership/clinical management will:**

|  | 1 - Strongly Disagree | 2 - Disagree | 3 - Neither Agree nor Disagree | 4 - Agree | 5 - Strongly Agree | Don't Know/Not Applicable |
| --- | --- | --- | --- | --- | --- | --- |
| a) propose a project that is appropriate and feasible |  |  |  |  |  |  |
| b) provide clear goals for improvement in patient care |  |  |  |  |  |  |
| c) establish a project schedule and deliverables |  |  |  |  |  |  |
| d) designate a clinical champion(s) for the project |  |  |  |  |  |  |

**13. Characteristics: The project clinical champion:**

|  | 1 - Strongly Disagree | 2 - Disagree | 3 - Neither Agree nor Disagree | 4 - Agree | 5 - Strongly Agree | Don't Know/Not Applicable |
| --- | --- | --- | --- | --- | --- | --- |
| a) accepts responsibility for the success of this project |  |  |  |  |  |  |
| b) has the authority to carry out the implementation |  |  |  |  |  |  |
| c) is considered a clinical opinion leader |  |  |  |  |  |  |
| d) works well with the intervention team and providers |  |  |  |  |  |  |

**14. Role: Senior leadership/clinical management/staff opinion leaders:**

|  | 1 - Strongly Disagree | 2 - Disagree | 3 - Neither Agree nor Disagree | 4 - Agree | 5 - Strongly Agree | Don't Know/Not Applicable |
| --- | --- | --- | --- | --- | --- | --- |
| a) agree on the goals for this intervention |  |  |  |  |  |  |
| b) will be informed and involved in the intervention |  |  |  |  |  |  |
| c) agree on adequate resources to accomplish the intervention |  |  |  |  |  |  |
| d) set a high priority on the success of the intervention |  |  |  |  |  |  |

**15. Role: The implementation team members:**

|  | 1 - Strongly Disagree | 2 - Disagree | 3 - Neither Agree nor Disagree | 4 - Agree | 5 - Strongly Agree | Don't Know/Not Applicable |
| --- | --- | --- | --- | --- | --- | --- |
| a) share responsibility for the success of this project |  |  |  |  |  |  |
| b) have clearly defined roles and responsibilities |  |  |  |  |  |  |
| c) have release time or can accomplish intervention tasks within their regular work load |  |  |  |  |  |  |
| d) have staff support and other resources required for the project |  |  |  |  |  |  |

**16. Style: The implementation plan for this intervention:**

|  | 1 - Strongly Disagree | 2 - Disagree | 3 - Neither Agree nor Disagree | 4 - Agree | 5 - Strongly Agree | Don't Know/Not Applicable |
| --- | --- | --- | --- | --- | --- | --- |
| a) identifies specific roles and responsibilities |  |  |  |  |  |  |
| b) clearly describes tasks and timelines |  |  |  |  |  |  |
| c) includes appropriate provider/patient education |  |  |  |  |  |  |
| d) acknowledges staff input and opinions |  |  |  |  |  |  |

**17. Style: Communication will be maintained through:**

|  | 1 - Strongly Disagree | 2 - Disagree | 3 - Neither Agree nor Disagree | 4 - Agree | 5 - Strongly Agree | Don't Know/Not Applicable |
| --- | --- | --- | --- | --- | --- | --- |
| a) regular project meetings with the project champion and team members |  |  |  |  |  |  |
| b) involvement of quality management staff in project planning and implementation |  |  |  |  |  |  |
| c) regular feedback to clinical management on progress of project activities and resource needs |  |  |  |  |  |  |
| d) regular feedback to clinicians on effects of practice changes on patient care/outcomes |  |  |  |  |  |  |

**18. Style: Progress of the project will be measured by:**

|  | 1 - Strongly Disagree | 2 - Disagree | 3 - Neither Agree nor Disagree | 4 - Agree | 5 - Strongly Agree | Don't Know/Not Applicable |
| --- | --- | --- | --- | --- | --- | --- |
| a) collecting feedback from patients regarding proposed/implemented changes |  |  |  |  |  |  |
| b) collecting feedback from staff regarding proposed/implemented changes |  |  |  |  |  |  |
| c) developing and distributing regular performance measures to clinical staff |  |  |  |  |  |  |
| d) providing a forum for presentation/discussion of results and implications for continued improvements |  |  |  |  |  |  |

**19. Resources: The following are available to make the selected plan work:**

|  | 1 - Strongly Disagree | 2 - Disagree | 3 - Neither Agree nor Disagree | 4 - Agree | 5 - Strongly Agree | Don't Know/Not Applicable |
| --- | --- | --- | --- | --- | --- | --- |
| a) staff incentives |  |  |  |  |  |  |
| b) equipment and materials |  |  |  |  |  |  |
| c) patient awareness/need |  |  |  |  |  |  |
| d) provider buy-in |  |  |  |  |  |  |
| e) intervention team |  |  |  |  |  |  |
| f) evaluation protocol |  |  |  |  |  |  |

**20. Evaluation: Plans for evaluation and improvement of this intervention include:**

|  | 1 - Strongly Disagree | 2 - Disagree | 3 - Neither Agree nor Disagree | 4 - Agree | 5 - Strongly Agree | Don't Know/Not Applicable |
| --- | --- | --- | --- | --- | --- | --- |
| a) periodic outcome measurement |  |  |  |  |  |  |
| b) staff participation/satisfaction survey |  |  |  |  |  |  |
| c) patient satisfaction survey |  |  |  |  |  |  |
| d) dissemination plan for performance measures |  |  |  |  |  |  |
| e) review of results by clinical leadership |  |  |  |  |  |  |

**End of survey.**

Thank you for your participation. Once you have submitted your survey, you will be given the option to download and print your responses as a PDF or Word document (through the toolbar located on the top of your browser). We recommend you do this so you can compare your responses over time.
