# Supplementary material for: Evaluation of the “Foundations in Knowledge Translation” training initiative: preparing end users to practice KT
Source: Implement Sci. 2018 Apr 25;13:63. doi: 10.1186/s13012-018-0755-4 (PMC5918493; doi:10.1186/s13012-018-0755-4)
Supplement: Supplementary file 5 — Baseline semi-structured interview guide for Foundations in KT participants. (DOCX 18 kb) [file 13012_2018_755_MOESM5_ESM.docx]

**“Foundations in KT” Participant Baseline Focus group Guide**

1. Introduction:

Hello Participant 1 and 2 [*insert names*], my name is XXX and I am a research coordinator/research assistant with the Knowledge Translation Program at St. Michael’s Hospital. Thank you very much for agreeing to participate in this focus group and taking the time to speak with me.

2. Purpose of study and focus group:

The goal of the Foundations project is to build knowledge and capacity in KT and to help participating teams plan and implement KT strategies/activities within their own projects with the support of mentors and communities of practice.

Prior to the start of the first workshop for the “Foundations of KT” course which will be coming up in April, we wanted to take this opportunity to speak with you as one of the participating teams to learn more about: your knowledge and experiences in the area of KT; your personal and professional learning goals; as well as the KT goals for your team projects. The information we discuss today will be used to inform the agenda for the upcoming workshop (Workshop 1) scheduled on April 9th and 10th in Vancouver.

3. Structure of the Focus group Process:

We will start off by briefly going over the terms of consent, after which I will ask for your verbal consent to participate in today’s focus group. Please note that a consent form has been mailed to you, regarding your participation in the Foundations of KT course and research component (i.e., the surveys and focus groups). Please review this form, sign, and return to St. Michael’s Hospital as indicated in the package; if you have already done so, thank you. Prior to starting this focus group, I will go over the terms of consent and ask for your verbal consent. Do you have any questions at this point? [*Address any concerns; if none, continue*].

During the focus group I will be asking you questions about your level of knowledge on KT, your learning goals, and any suggestions you have regarding the content of the education intervention. Some of the questions will be posed in turn to each of you (I will call your name to prompt you to respond and then turn it over to your teammate(s)); other questions I will open up for any or all members to respond to as they are more general. The focus group should take approximately 30 minutes to complete but no longer than 45 minutes.

The results of today’s focus group will help us to better understand your learning needs and inform the education intervention that you will be taking part in. Do you have any questions at this point? [*Address any concerns; if none, continue*].

4. Consent

*Please complete required fields based on participant responses.*

Have you received the Foundations of KT consent forms that were mailed to you?

| Participant X: [*insert full name name*] | Yes/No/Not applicable |
| --- | --- |
| Participant 1: |  |
| Participant 2: |  |
| Participant 3: |  |

If yes, have you reviewed, signed, and returned to St. Michael’s Hospital using the return envelope provided?

If no, please review, sign, and return a signed copy of consent form to St. Michael’s Hospital using the return envelope provided. Two copies have been provided; please retain one copy for your own records.

| Participant X: [*use* *initials from above*] | Status of Consent Form (e.g., mailed including date if provided, reviewed/signed but not mailed, not yet reviewed/signed or mailed, not yet received) |
| --- | --- |
| Participant 1: |  |
| Participant 2: |  |
| Participant 3: |  |

*Outline the terms of verbal consent.*

I will now go over the terms of consent:

Your participation in this study is voluntary.

You can choose to not participate or you may withdraw at any time, even after the focus group has started.

This focus group is confidential; only I will know your identity.

The focus group will be recorded.

The audiotape will be transcribed and names will be removed as it is being transcribed.

Once the transcribed information has been assessed for accuracy by the study coordinator, the audiotape will be erased.

The focus group will be analyzed by an independent analyst who will not know your identity.

The results will be aggregated and reported anonymously. The results may be used in presentations and publications.

If you would like a report of the results, we can provide you with a summary when analysis is complete.

At this point, do you have any questions?

I will now get started by turning on the recorder and asking you, in turn, to state your verbal consent to participate in today’s focus group.

Today is [*insert date*] and I am focus grouping Foundations Team X [will be assigned, insert here]; *AND if applicable state group number: Group Y [will be assigned, insert here].* Participant 1 [*insert name*], do you consent to being focus grouped and recorded today? Repeat for all other participants on the call.

5. Start the focus group

|  | Focus group Question | Individual versus Team Questions  Individual = ask each participant on the call to respond in turn.  Team= open up question to all participants on the call. |
| --- | --- | --- |
| 1. | How would you define KT in your own words? | Individual |
| 2. | Do you know any KT frameworks  If yes, can you name/describe them? | Individual |
| 3. | What do you feel are your key knowledge gaps in the area of knowledge translation? | Individual |
| 4. | What are your learning goals regarding knowledge translation?  How motivated are you to meet these goals? | Individual |
| 5. | What are the KT goals for your project? | Team |
| 6. | Do you feel that there are any challenges to meet your KT goals currently? | Team |
| 7. | Do you feel that you have the resources to meet these goals?  Can you describe the resources available to you? | Team |
| 8. | What changes (or impacts) do you hope to see as a result of meeting these KT goals for your project? | Team |
| 9. | What do you think are the benefits of learning about KT, if any? | Individual |
| 10. | Will learning more about KT help you in your professional role? Why/why not? | Individual |
| 11. | What are you hoping to see on the agenda for our first workshop in April? | Individual |

Wrap Up

Thank you for your time today. It was great to connect before the upcoming workshop and learn more about your KT project goals and individual learning needs. Rami and Caitlyn will be in touch with you shortly to provide additional details about the April workshop including the recommended pre-readings, the workshop agenda and other important details.
