# Supplementary material for: Evaluation of the “Foundations in Knowledge Translation” training initiative: preparing end users to practice KT
Source: Implement Sci. 2018 Apr 25;13:63. doi: 10.1186/s13012-018-0755-4 (PMC5918493; doi:10.1186/s13012-018-0755-4)
Supplement: Supplementary file 6 — 6, 12, 18, and 24 month semi-structured interview guide for Foundations in KT participants. (DOCX 24 kb) [file 13012_2018_755_MOESM6_ESM.docx]

**Foundations in KT Participants Follow-Up Focus group Guide (6, 12, 18, 24 months)**

1. Introduction:

Hello Participant 1 and 2 [*insert names*], my name is XXX and I am a research coordinator/research assistant with the Knowledge Translation Program at St. Michael’s Hospital. Thank you very much for agreeing to participate in this focus group and taking the time to speak with me.

2. Purpose of study and focus group:

As you may recall, the goal of the Foundations of KT project is to build knowledge and capacity in KT and to help participating teams plan and implement KT strategies/activities for their own projects with the support of mentors and communities of practice.

Following workshop #1 (hosted in April 2014) and prior to workshop #2 (scheduled for October 2014), we wanted to take this opportunity to speak with you as one of the participating teams to discuss your experience with the course to date as well as your continued learning goals, project accomplishments and challenges, and needs moving forward. The information we discuss today will be considered for ongoing improvements in the course design and to inform the agenda for the upcoming workshop (workshop #2) scheduled on October 22nd in Vancouver.

3. Structure of the Focus group Process:

We will start off by briefly going over the terms of consent, after which I will ask for your verbal consent to participate in today’s focus group. The terms of consent outlined today were also included in the hard copy of the consent form that you completed at the start of the course. Do you have any questions at this point? [*Address any concerns; if none, continue*].

During the focus group, I will be asking you questions about your experiences with the Foundations of KT course to date (e.g., things you liked versus things that could be improved about workshop #1, the Canvas platform, support from project team); your ongoing learning goals; and project accomplishments and challenges so far. Some of the questions will be posed in turn to each of you (I will call your name to prompt you to respond and then turn it over to your teammate(s)); other questions I will open up for any or all members to respond to as they are more general. The focus group will take approximately 45 minutes to complete but no longer than 1 hour.

The results of today’s focus group will help us to evaluate the course components, better understand your learning needs, and inform the upcoming workshop that you will be taking part in. Do you have any questions at this point? [*Address any concerns; if none, continue*].

4. Consent

*Outline the terms of verbal consent.*

I will now go over the terms of consent:

Your participation in this study is voluntary.

You can choose to not participate or you may withdraw at any time, even after the focus group has started.

This focus group is confidential; only I will know your identity.

The focus group will be recorded.

The audiotape will be transcribed and names will be removed as it is being transcribed.

Once the transcribed information has been assessed for accuracy by the study coordinator, the audiotape will be erased.

The focus group will be analyzed by an independent analyst who will not know your identity.

The results will be aggregated and reported anonymously. The results may be used in presentations and publications.

If you would like a report of the results, we can provide you with a summary when analysis is complete at the end of the 24-month project period.

At this point, do you have any questions?

I will now get started by turning on the recorder and asking you, in turn, to state your verbal consent to participate in today’s focus group. [TURN ON AUDIO RECORDER]

Today is [*insert date*] and I am focus grouping Foundations Team X [will be assigned, insert here]; *AND if applicable state group number: Group Y [will be assigned, insert here].* [*insert name of participant*], do you consent to being focus grouped and recorded today? *Repeat for all other participants on the call.*

5. Start the focus group

|  | Focus group Question | Individual versus Team Questions  Individual = ask each participant on the call to respond in turn.  Team= open up question to all participants on the call. |
| --- | --- | --- |
| Questions about experiences with the Foundations of KT Course | | |
| 1. | On a scale from 1 to 5, where 1 is not at all satisfied and 5 is extremely satisfied, how would you rate your overall level of satisfaction with the Foundations of KT course so far?  *Please elaborate on your rating.* | Individual |
| 2. | What are your thoughts on the format of the Foundations of KT course so far?  *What would be useful to you going forward regarding the format of the intervention?* | Team |
| 3. | What are your thoughts on the content of the course so far?  *What would be useful to you going forward regarding the content of the intervention?* | Team |
| 4. | What do you think about the Canvas platform?  Do you use Canvas? How often?  What do you find helpful about it?  How could it be improved? | Team |
| 5. | Do you feel that the course has enabled you to meet your KT learning goals to date?  *Why/why not?* | Individual |
| 6. | Do you feel that the course has enabled you to meet your KT project goals?  *Why/why not?* | Team |
| 7. | Do you feel confident in your ability to be able to plan and participate in KT projects at your institution? Why/why not?  If yes, do you feel that the education intervention helped build this confidence?  If no, what could have been done in the education intervention to help increase your confidence in your abilities? | Individual |
| 8. | Can you describe your commitment to engaging in KT?  Has your commitment level been influenced by participating in this education intervention?  What would help to keep you engaged? | Individual |
| Questions about project implementation at participants’ organizations | | |
| 9. | Can you describe the progress you have made with your implementation project to date? | Team |
| 10. | What do you think are the main barriers or challenges to implementing your project? The facilitators? | Team |
| 11. | Can you describe the attitudes of other staff members towards your implementation project? | Team |
| 12. | What is the capacity at your institution to be able to implement your project?  Do you feel that you have enough resources to be able to implement the project?  Are there systems in place at your institution to enable the sustainability of your project? | Team |
| 13. | Can you describe your institution’s level of commitment to KT?  Do you see KT as a priority at your institution?  Is your specific project a priority at your institution? | Team |
| 14. | What are you hoping to see on the agenda for our second workshop in October? | Individual |
| *Do you have any additional comments that you would like to add?* | | Team |

Wrap Up

Thank you for your time today. It was great to connect before the upcoming workshop and hear about your experiences with the Foundations of KT course so far. Caitlyn and Shusmita will be in touch with you over the coming weeks to provide additional details about Workshop #2 including the workshop agenda and other important details.

Decision Maker Follow-Up Focus group Guide (6, 12, 18, 24 months)

1. Introduction:

Hello [*insert name*], my name is XXX and I am a research coordinator/research assistant with the Knowledge Translation Program at St. Michael’s Hospital. Thank you very much for agreeing to participate in this focus group and taking the time to speak with me.

2. Purpose of study and focus group:

As you may recall, the goal of the Foundations of KT project is to build knowledge and capacity in KT and to help participating teams plan and implement KT strategies/activities for their own projects with the support of mentors and communities of practice.

We wanted to take this opportunity to obtain feedback from you as the identified decision maker partner for one of our participating teams and hear about your thoughts on the team’s implementation project, your institutional capacity for implementation, and barriers and facilitators to implementation.

3. Structure of the Focus group Process:

We will start off by briefly going over the terms of consent, after which I will ask for your verbal consent to participate in today’s focus group. The terms of consent outlined today were also included in the hard copy of the consent form that you completed at the start of the project. Do you have any questions at this point? [*Address any concerns; if none, continue*].

During the focus group I will be asking you questions about your perceptions of the team’s project, KT and implementation priorities and capacity at your institution and barriers and facilitators to implementation and sustainability. The focus group will take approximately 30 minutes to complete but no longer than 45 minutes.

The results of today’s focus group will help us to evaluate the course components and understand the implementation context for the team’s project. Do you have any questions at this point? [*Address any concerns; if none, continue*].

4. Consent

*Outline the terms of verbal consent.*

I will now go over the terms of consent:

- Your participation in this study is voluntary.
- You can choose to not participate or you may withdraw at any time, even after the focus group has started.
- This focus group is confidential; only I will know your identity.
- The focus group will be recorded.
- The audiotape will be transcribed and names will be removed as it is being transcribed.
- Once the transcribed information has been assessed for accuracy by the study coordinator, the audiotape will be erased.
- The focus group will be analyzed by an independent analyst who will not know your identity.
- The results will be aggregated and reported anonymously. The results may be used in presentations and publications.
- If you would like a report of the results, we can provide you with a summary when analysis is complete at the end of the 24-month project period.

At this point, do you have any questions?

I will now get started by turning on the recorder and asking you to state your verbal consent to participate in today’s focus group. [TURN ON AUDIO RECORDER]

Today is [*insert date*] and I am focus grouping Foundations Decision Maker X [ID will be assigned, insert here]. Do you consent to being focus grouped and recorded today?

5. Start the focus group

|  | **Focus group Question** |
| --- | --- |
| 1. | Can you briefly describe your role and relationship to the project [*referring to the project being led by participating team in the Foundations of KT course*]? |
| 2. | Can you describe the project [*referring to the project being led by participating team in the Foundations of KT course]* from your perspective?   - What are your impressions and thoughts on this project? |
| 3. | What is your past experience with implementation at your organization/institution?   - What do you think will be different this time (if anything)? |
| 4. | What is the capacity at your organization/institution to be able to implement this project?   - Do you feel that your organization/institution has enough resources to be able to implement the project successfully? - If not, how can capacity be built at your organization/institution for successful implementation? |
| 5. | Can you describe your institutional level of commitment to KT?   - Do you see KT as a priority at your organization/institution? - Is the specific implementation project a priority at your organization/institution? - Are there competing priorities at your organization/institution? Can you describe these? |
| 6. | Can you describe the attitudes of other staff members towards the implementation project?   - Do you feel that staff members influence each other greatly in their attitudes and practices? |
| 7. | What do you think are the main barriers or challenges to implementing the project? |
| 8. | What do you think are the main facilitators or opportunities for implementing the project? |
| 9. | Are there systems in place at your organization/institution to enable the sustainability of the project?   - What strategies/activities can help to maintain the changes that will be/have been made by the project? - Are there any systems or initiatives in place that can help the project scale up or spread? |
| Do you have any suggestions or additional comments that you would like to add? | |

Wrap Up

Thank you for your time today.
