# Supplementary material for: Evaluation of the “Foundations in Knowledge Translation” training initiative: preparing end users to practice KT
Source: Implement Sci. 2018 Apr 25;13:63. doi: 10.1186/s13012-018-0755-4 (PMC5918493; doi:10.1186/s13012-018-0755-4)
Supplement: Supplementary file 7 — 6, 12, 18, and 24 month semi-structured interview guide for DMPs. (DOCX 24 kb) [file 13012_2018_755_MOESM7_ESM.docx]

**Interview Guide for Decision Maker Partners (~45-60 minutes)**

1. Introduction:

Hello [*insert name*], my name is XXX and I am a research coordinator/research assistant with the Knowledge Translation Program at St. Michael’s Hospital. Thank you very much for agreeing to participate in this interview and taking the time to speak with me.

2. Purpose of study and interview:

As you may recall, the goal of the Foundations of KT project is to build knowledge and capacity in KT and to help participating teams plan and implement KT strategies/activities for their own projects with the support of mentors and communities of practice.

We wanted to take this opportunity to obtain feedback from you as the identified decision maker partner for one of our participating teams and hear about your thoughts on the team’s implementation project, your institutional capacity for implementation, and barriers and facilitators to implementation.

3. Structure of the Interview Process:

We will start off by briefly going over the terms of consent, after which I will ask for your verbal consent to participate in today’s interview. The terms of consent outlined today were also included in the hard copy of the consent form that you completed at the start of the project. Do you have any questions at this point? [*Address any concerns; if none, continue*].

During the interview I will be asking you questions about your perceptions of the team’s project, KT and implementation priorities and capacity at your institution and barriers and facilitators to implementation and sustainability. The interview will take approximately 30 minutes to complete but no longer than 45 minutes.

The results of today’s interview will help us to evaluate the course components and understand the implementation context for the team’s project. Do you have any questions at this point? [*Address any concerns; if none, continue*].

4. Consent

*Outline the terms of verbal consent.*

I will now go over the terms of consent:

- Your participation in this study is voluntary.
- You can choose to not participate or you may withdraw at any time, even after the interview has started.
- This interview is confidential
- The interview will be recorded.
- The audiotape will be transcribed and names will be removed as it is being transcribed.
- Once the transcribed information has been assessed for accuracy by the study coordinator, the audiotape will be erased.
- The interview will be analyzed by an independent analyst who will not know your identity.
- The results will be aggregated and reported anonymously. The results may be used in presentations and publications.
- If you would like a report of the results, we can provide you with a summary when analysis is complete at the end of the 24-month project period.

At this point, do you have any questions?

I will now get started by turning on the recorder and asking you to state your verbal consent to participate in today’s interview. [TURN ON AUDIO RECORDER]

Today is [*insert date*] and I am interviewing Foundations Decision Maker X [ID will be assigned, insert here]. Do you consent to being interviewed and recorded today?

FLIP PAGE FOR INTERVIEW QUESTIONS

5. Start the interview

|  | **Interview Question** |
| --- | --- |
| 1. | Can you briefly describe your role in/relationship to the project [*referring to the project being led by participating team in the Foundations of KT course*]?   - Can you describe your role/position within the organization/institution? |
| 2. | Can you describe the project [*referring to the project being led by participating team in the Foundations of KT course]* from your perspective?   - What are your impressions and thoughts on this project? |
| 3. | What is your past experience with implementation at your organization/institution?   - What do you think will be different this time (if anything)? |
| 4. | What is the capacity at your organization/institution to be able to implement this project?   - Do you feel that your organization/institution has enough resources to be able to implement the project successfully? - If not, how can capacity be built at your organization/institution for successful implementation? |
| 5. | Can you describe your institutional level of commitment to KT?   - Do you see KT as a priority at your organization/institution? - Is the specific implementation project a priority at your organization/institution? - Are there competing priorities at your organization/institution? Can you describe these? |
| 6. | Can you describe the attitudes of other staff members towards the implementation project?   - Do you feel that staff members influence each other greatly in their attitudes and practices? |
| 7. | What do you think are the main barriers or challenges to implementing the project? |
| 8. | What do you think are the main facilitators or opportunities for implementing the project? |
| 9. | Are there systems in place at your organization/institution to enable the sustainability of the project?   - What strategies/activities can help to maintain the changes that will be/have been made by the project? - Are there any systems or initiatives in place that can help the project scale up or spread? |
| Do you have any suggestions or additional comments that you would like to add? | |

Wrap Up

Thank you for your time today.
